# A MOBILE HOME HEALTH CARE PHYSICIAN FOR OLDER PERSONS WITH EXTENSIVE HEALTH CARE NEEDS

**DOI:** 10.1093/geroni/igad104.2253

**Published:** 2023-12-21

**Authors:** Lina Emmesjö, Jenny Hallgren, Anna Dahl Aslan, Catharina Gillsjö (Gillsjo)

**Affiliations:** University of Skövde, Skövde, Vastra Gotaland, Sweden; University of Skövde, Skövde, Vastra Gotaland, Sweden; University of Skövde, Skövde, Vastra Gotaland, Sweden; University of Skövde, Skövde, Vastra Gotaland, Sweden

## Abstract

**Background:**

The rapidly increasing older population with extensive care needs has shifted health care from institutions to the older person’s home. A cross-organisational integrated care model was created by health care authorities to meet these challenges, the Mobile integrated care model. The Mobile integrated care model with a home health care physician is a collaboration between regional and municipal health care, working in the patients’ home.

**Methods:**

Semi-structured interviews with patients, next of kin and health care professionals.

**Results:**

The home was described by all as the best place to provide health care to these patients, creating safety and increasing autonomy for the patients. The health care professionals found trust in working together as a team, but struggled because of the divided organizations. Patients and next of kin found the Mobile integrated care model to be hierarchic, where the structure sometimes improved participation, and at other times prevented it.

**Conclusion:**

All participant groups emphasized that there was a need for more time for the health care personnel to spend with the patients. Furthermore, the patients and next of kin longed for a personal contact and being able to form a relationship with the health care personnel. The health care professionals found being employed by separate organizations as a challenge, where divided documentation systems and lack of equipment hindered the work.

